# SARS-CoV-2 Transmission Risk Model in an Urban Area of Mexico, Based on GIS Analysis and Viral Load

**DOI:** 10.3390/ijerph19073840

**Published:** 2022-03-24

**Authors:** Victor Wagner Barajas-Carrillo, Carlos Eduardo Covantes-Rosales, Mercedes Zambrano-Soria, Lucia Amapola Castillo-Pacheco, Daniel Alberto Girón-Pérez, Ulises Mercado-Salgado, Ansonny Jhovanny Ojeda-Durán, Erica Yolanda Vázquez-Pulido, Manuel Iván Girón-Pérez

**Affiliations:** Laboratorio Nacional de Investigación para la Inocuidad Alimentaria (LANIIA) Unidad Nayarit, Universidad Autónoma de Nayarit, Tepic 63000, Nayarit, Mexico; wagner.laniia@uan.edu.mx (V.W.B.-C.); carlos.covantes@gmail.com (C.E.C.-R.); mzambrano.laniia@gmail.com (M.Z.-S.); trabajolucia25@gmail.com (L.A.C.-P.); daniel.giron@uan.edu.mx (D.A.G.-P.); ulises.mercado.salgado@gmail.com (U.M.-S.); ansonnyjho@gmail.com (A.J.O.-D.); erica.vazquez@uan.edu.mx (E.Y.V.-P.)

**Keywords:** Mexico, COVID-19, GIS, heatmap, risk, viral load, vaccination, qRT-PCR, kernel density, SARS-CoV-2

## Abstract

The COVID-19 pandemic highlighted health systems vulnerabilities, as well as thoughtlessness by governments and society. Due to the nature of this contingency, the use of geographic information systems (GIS) is essential to understand the SARS-CoV-2 distribution dynamics within a defined geographic area. This work was performed in Tepic, a medium-sized city in Mexico. The residence of 834 COVID-19 infected individuals was georeferenced and categorized by viral load (Ct). The analysis took place during the maximum contagion of the first four waves of COVID-19 in Mexico, analyzing 158, 254, 143, and 279 cases in each wave respectively. Then heatmaps were built and categorized into five areas ranging from very low to very high risk of contagion, finding that the second wave exhibited a greater number of cases with a high viral load. Additionally, a spatial analysis was performed to measure urban areas with a higher risk of contagion, during this wave this area had 19,203.08 km^2^ (36.11% of the city). Therefore, a kernel density spatial model integrated by meaningful variables such as the number of infected subjects, viral load, and place of residence in cities, to establish geographic zones with different degrees of infection risk, could be useful for decision-making in future epidemic events.

## 1. Introduction

Coronavirus Disease-19 (COVID-19), produced by the severe acute respiratory syndrome coronavirus 2 (SARS-CoV-2), has become a global pandemic and threat to public health [1]. Since the COVID-19 outbreak in Hubei China, this epidemic has displayed a three-wave pattern in several countries [2]. The symptomatology of SARS-CoV-2 is highly variable; however, common symptoms are observed in other infectious diseases. Patients exhibit fever, dry cough, loss of smell and/or taste, gastrointestinal complaints, headache, muscle pain, and chest pain, in the most severe cases, respiratory distress and inability to speak or move, even death [3,4].

Globally, countries implemented restrictive and sanitary measures to control the rate of infection, however, these measures due to haste and ignorance have caused significant economic losses. For this reason, throughout the months of the pandemic, due to the demands of the population, governments have relaxed health measures, which has led to an increase in cases and the appearance of episodes of greater contagion known generally as outbreaks or waves of COVID-19 [5].

In the case of Mexico, on 26 January 2021, official data showed that the number of confirmed COVID-19 cases worldwide reached 360.819.888, of which 4.730.669 were from Mexico. This country has faced a four-wave pattern of laboratory-confirmed COVID-19 cases; the first wave occurred in summer 2020, the second in January 2021, the third appeared in August 2021, and the fourth started in January 2022 [6].

A very important factor but rarely considered for the transmission of the SARS-CoV-2 virus is the value of the viral load that each individual presents during infection [7], as well as for transmission by air in closed spaces [8]. Thus, high viral load has been associated with greater severity in other coronavirus outbreaks, as previously reported during the SARS epidemic in 2003 [9]. This same relationship has been seen in other respiratory viral diseases (rhinovirus), presenting respiratory symptoms as well as aggravated inflammatory processes in patients with high viral loads [10]. Thus, in the case of the pandemic caused by the SARS-CoV-2 virus, low Ct values indicate a high viral load (Ct value is inversely proportional to the number of viral particles) in infected people, so these individuals facilitate the spread of the virus in a community.

In this way, the viral load present in each person depends on various factors, among which the immunocompetence of everyone stands out. Subjects with a robust and adequate immune response can effectively control the infection and will present low viral loads, and are even usually asymptomatic subjects. In contrast, people with greater susceptibility or less immunocompetence will present high viral loads during infection and a greater probability of developing severe COVID-19. In this sense, vaccination is one of the most effective strategies to control the pandemic.

An effective way to estimate the viral load presented by each infected subject is through a quantitative or semi-quantitative qRT-PCR analysis. The time necessary to amplify the viral genetic material in each biological sample is inversely proportional to the cycle threshold (Ct) for virus amplification. A Ct value < 29 is indicative of a high viral load, while a Ct > 34 means a low viral load. Thus, the Ct value is a parameter that can be used to assess the risk of community transmission [11].

Geographic Information Systems (GIS) are the set of tools that systematically organize and relate large amounts of properly georeferenced information, which once interconnected provides a model or response to some phenomenon of interest. During the COVID-19 pandemic, the use of GIS has been crucial for understanding the dynamics of the spread of the SARS-CoV-2 virus. In this sense, our research group proposes a transmission risk assessment model that considers the viral load of people diagnosed with the COVID-19 disease in combination with the proximity of their homes in the same period. Thus, the objective of this article was to implement a kernel density model, which combined the values of viral load (expressed in Ct), the number of people infected with SARS-CoV-2, as well as their place of residence in the city of Tepic, Mexico; to establish geographic zones with different degrees of infection risk within an urban zone during the maximum peaks of the four waves presented in Mexico.

## 2. Materials and Methods

### 2.1. Study Area

Tepic is the capital of the state of Nayarit, Mexico, it is in the central part of the state and is located at the extreme geographic coordinates 21°51′ and 21°24′, north latitude and 104°34′ and 105°05′ west longitude. Tepic concentrates 34.5% of the total population of Nayarit, which is about 371,387 people within the urban area, with a population density of 265.8 inhabitants per km². The population is distributed almost evenly with 51.4% women and 48.6% men. Currently, the state capital has 133,816 homes, of which 126,186 are habited, with an average of 3.3 occupants per home, considering these numbers the average occupants per room is 0.8 [12].

### 2.2. Study Design and Participants

Outpatients who attended qRT-PCR testing for SARS-CoV-2 at the LANIIA-UAN laboratory (laboratory approved by the Mexican health authorities) were prospectively evaluated. All outpatients with laboratory-confirmed COVID-19 disease were selected during the dates that the four waves of COVID-19 took place in the state of Nayarit, Mexico. Based on official Mexican data, the peak dates of the SARS-CoV-2 pandemic in the state of Nayarit, Mexico, were detected. The maximum peak of the pandemic in the first, second, third, and fourth waves was 1 August 2020; 21 January 2021, 19 August 2021, and 17 January 2022, respectively. Then, all data recorded (age, sex, and viral load (expressed in Ct)) ±15 days the date of the maximum peaks of the four waves of COVID-19 (15 July to 14 August 2020; 6 January to 5 February 2021; 4 August to 3 September 2021; and 3 January to 2 February 2022, corresponding to the first, second, third and fourth waves of COVID-19), from ambulatory patients who attended the molecular diagnosis of SARS-CoV-2 at the LANIIA laboratory facilities, whose result was positive, were selected.

All patients signed informed consent before sampling, clinical data, and demographic information were also collected. This study was approved by the local bioethics commission registry number (CEBN/03/20). Subjects were queried to avoid eating food, drinking water, and brushing teeth at least 4 h before sampling for sample collection.

### 2.3. Swabbing

For swab sampling, a flexible swab was passed through each subject’s nostril reaching the nasopharynx. Another flexible swab was introduced through the mouth to reach the oropharynx. Both swabs were placed in the mucosa while gently circling for some seconds, then removed while rotating and placed in 2.5 mL of the sterile VTM. Sterile VTM (pH 7.10) was prepared with Hank’s balanced salt solution (HBSS) (ThermoFisher, Scientific, Cat n° 14190, Cleveland, OH, USA) supplemented with gentamicin sulfate (4 mg/mL) (ThermoFisher Scientific, Cat n° 15750078) penicillin/streptomycin (50,000 U/50,000 mg/mL) (ThermoFisher Scientific; Cat n° 15140148), amphotericin B (0.4 mg/mL) (ThermoFisher Scientific; Cat n° 15290018) and bovine serum albumin (5%) (ThermoFisher Scientific; Cat n° 15561020).

### 2.4. qRT-PCR Procedure

Methods were validated by Mexican health authorities (Mexican Health Ministry—Secretaría de Salud de México—, and InDRE—). All reagents, kits, and procedures were approved by these authorities. Inactivation and total RNA extraction were performed with RNA extraction by QIAmp Viral RNA Mini Kit (Qiagen, Cat No./ID: 1020953 USA, Germantown, TN, USA) with 140 μL of VTM from swabbing. The qRT-PCR procedure was performed according to the Berlin protocol with modifications [13]. Briefly, one-step qRT-PCR was performed with StarQ One-Step qRT-PCR (Qiagen, Cat No./ID: 210210, USA, Germantown kit), with the extracted RNA from samples. The following oligonucleotides were used for the molecular detection of the SARS-CoV-2 E gene; E_Sarbeco_Forward: ACAGGTACGTTAATAGTTAATAGCGT, E_Sarbeco_Reverse: ATATTGCAGCAGTACGCACACA, TaqMan probe E_Sarbeco_P1: FAMCACTAGCCATCCTTACTGCGCTTCG-BBQ, and as a control, RNase P gene; RNAseP Forward: AGATTTGGACCTGCGAGCG, RNAseP Reverse, GAGCGGCTGTCTCCACAAGT, TaqMan probe RNAseP P1, FAMTTCTGACCTGAAGGCTCTG CGCG-BHQ1 [13,14]. qRT-PCR was performed with 5 μL (70 ng/μL) of extracted RNA in a total 25 μL reaction. All samples were analyzed with a 7500 Real-Time PCR System (Applied Biosystems) with the following protocol: 50 °C for 15 min, 95 °C for 2 min, and then 45 cycles of 95 °C for 15 s, 82 °C and 60 °C for 30 s. In all cases, human gene (RNAseP) amplification was used as an internal control, and samples were considered as positive if the number of cycles needed for the fluorescent signal to cross the cycle threshold, known as Ct value, was equal to or lower than 38.

### 2.5. GIS Methodology

A database was designed with the information collected from patients diagnosed with the SARS-CoV-2 virus in our laboratory. The data included in the database were divided into four waves, which were delimited, considering the day on which the maximum peak of positive cases occurred ± 15 days; thus, the first wave comprised the period from 15 July to 14 August 2020; the second wave from 6 January to 5 February, the third wave from 4 August to 3 September 2021, and the fourth wave from 3 January to 2 February 2022. The information collected from each infected individual was the following data: address, zip code, age, sex, date of diagnosis, and Ct value (viral load).

Collected data was analyzed through GIS with the QGIS 3.20.2 ‘Odense’ software, where each positive case was georeferenced and vector shapefile points were generated, one for each wave, the positive cases were categorized according to the Ct value of each case (Ct < 29, high viral load; Ct = 30–33 mean viral load; Ct = 34–37 low viral load). This information was completed with the most up-to-date and official data from the National Institute of Statistics, Geography, and Informatics (INEGI-Mexico), as well as the shapefile vector files of the urban Basic Geostatistical Area (AGEB) and city block of the city of Tepic. With the above, a heatmap was generated for each wave of COVID-19, which considered the distance matrix and definition of the radius between infected individuals and the value of the viral load. All the layers used and produced in this work were adjusted to the reference system: EPSG: 32613, Datum: WGS 84, with a projection: UTM zone 13N.

### 2.6. Heatmaps

The heatmaps were built from a vector file for each wave. The mean and standard deviation data of the proximity radius were obtained according to the mathematical expression described in Rizzatti and co-workers, 2020. The value of the proximity criterion used for each radius was 1022.82 ± 57.07 m. Once this value was obtained, it proceeded to generate the maps with the QGIS software process toolbox (one map per wave). In which, the pixel size was 1. In advanced parameters, the Ct attribute was chosen for the weight from the field. To finalize the kernel shape, the quartic type of option was used, where the algorithm weighted the proximity between the points with greater weight and gave less weight the farther one point was from another [15]. The options radius from the field and output value scaling were left unchecked. Once the raster type file of the raw heat map was obtained, the raster layer with the shape of the urban area was extracted and, finally, the raster was reclassified to obtain the distribution categories among the positive cases.

### 2.7. Statistical Analysis

The database of positive cases for SARS-CoV-2 in our laboratory was processed in MS Excel^®^ and SigmaPlot 11.0 statistical software^®^ for analysis, with which the range, mean, and percentages were obtained. To measure the extent of the risk areas generated in the heatmap, the field calculator option in the QGIS was used.

## 3. Results

The total sum of positive subjects during the four-time periods evaluated (first, second, and third wave) was 834 cases (389 males and 445 females). During the first wave, 158 cases were tested (81 males and 77 females), during the second wave, 254 cases were tested (114 males and 140 females), in the third wave were 143 cases (64 males and 79 females) while for the fourth wave there were 279 cases (130 males and 149 females) Table 1.

**Figure 1:**
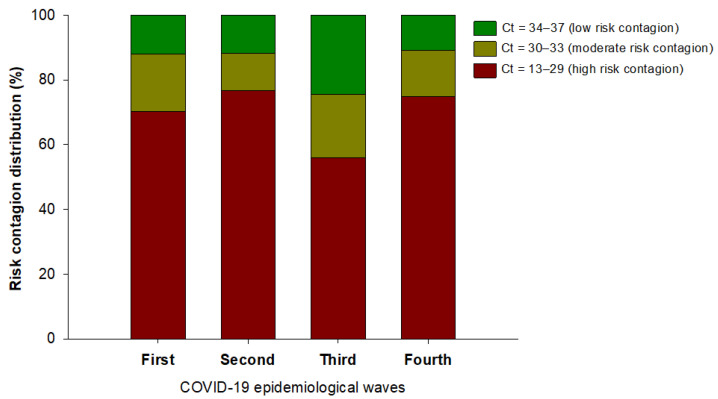
Percentages of people categorized (Ct < 29 high viral load, Ct 30–33 medium viral load, Ct 34–37 low viral load. When the Ct value is >38 it is considered a negative result for the detection of SARS-CoV-2 according to the value of Ct (viral load) during the four waves of COVID-19 analyzed.

For the categorization of positive cases, the criterion used was based on that described by Oba et al., 2021, who established the risk stratification parameters based on Ct values determined by qRT-PCR. Figure 1 shows the accumulated values of subjects and their percentage according to the viral load in each wave of COVID-19.

### 3.1. Heatmaps and Risk Area Analysis

**Figure 2:**
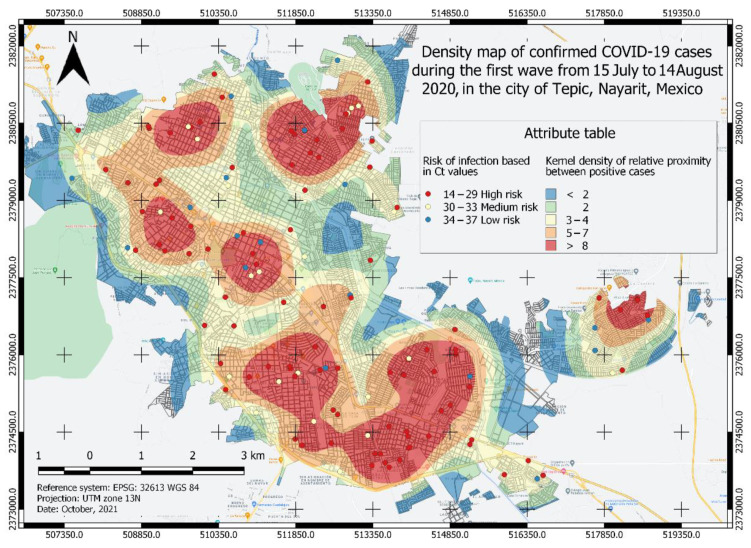
Density map of confirmed COVID-19 cases in the first wave from 15 July to 14 August 2020, in the city of Tepic, Nayarit, Mexico. The different areas classified by color indicate the level of risk of probability of contagion of the COVID-19 disease and each point corresponds to a confirmed case categorized by its Ct value.

**Figure 3:**
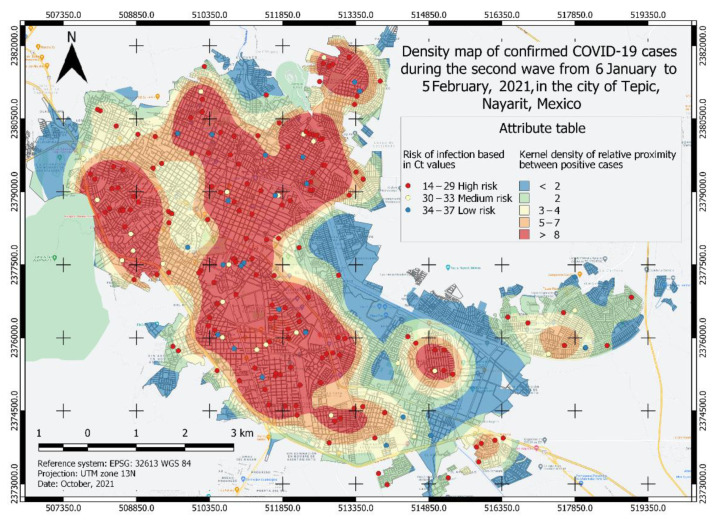
Density map of confirmed COVID-19 cases in the second wave from 6 January to 5 February 2021, in the city of Tepic, Nayarit, Mexico. The different areas classified by color indicate the level of risk of probability of contagion of the COVID-19 disease and each point corresponds to a confirmed case categorized by its Ct value.

**Figure 4:**
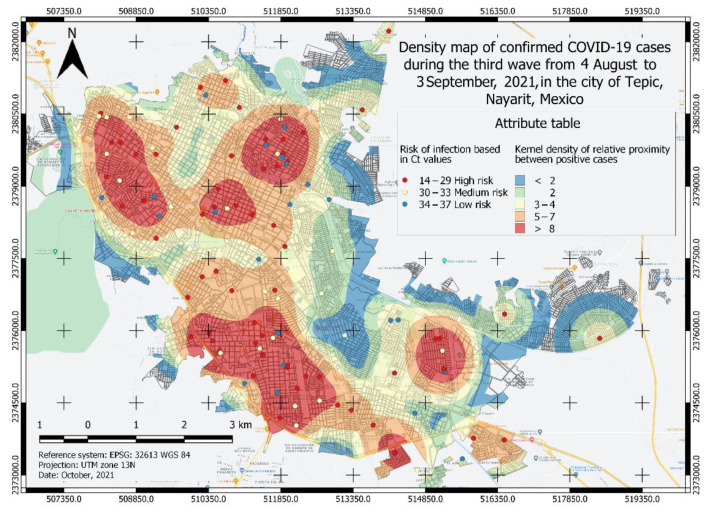
Density map of confirmed COVID-19 cases in the third wave from 4 August to 3 September 2021, in the city of Tepic, Nayarit, Mexico. The different areas classified by color indicate the level of risk of probability of contagion of the COVID-19 disease and each point corresponds to a confirmed case categorized by its Ct value.

**Figure 5:**
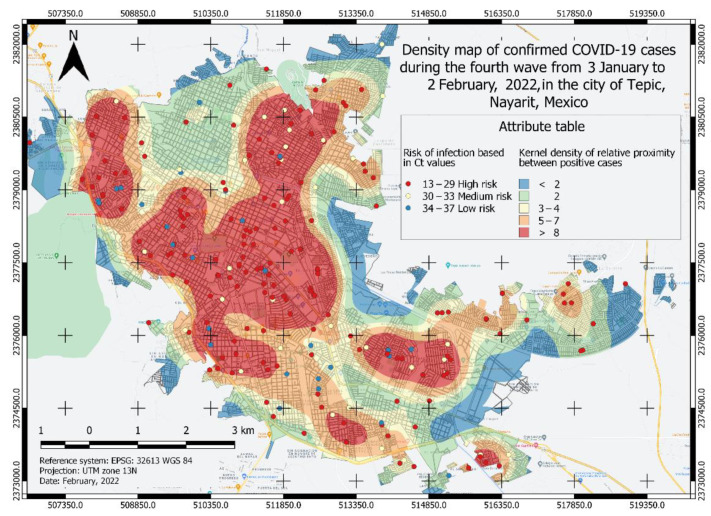
Density map of confirmed COVID-19 cases in the fourth wave from 3 January to 2 February 2021, in the city of Tepic, Nayarit, Mexico. The different areas classified by color indicate the level of risk of probability of contagion of the COVID-19 disease and each point corresponds to a confirmed case categorized by its Ct value.

A total of four heatmaps of kernel density of positive cases for COVID-19 were generated (Figure 2, Figure 3, Figure 4 and Figure 5), which were categorized into five classes from lower to a higher risk of contagion according to the proximity between them and for its Ct value. The first class was classified as a zone of very low probability of contagion delimited in blue, which corresponded to a proximity density of fewer than 2 cases. The next class was classified as a zone of low probability of contagion marked in green, which corresponded to a proximity density of 2 cases. Third, an area of a medium probability of contagion was delimited, which was identified in yellow with a proximity of 3–4 positive cases. The next class corresponded to the area marked in orange that was identified with the area of high risk of contagion. with 5–7 positive cases close to each other. The fifth class identified in red corresponds to an area of a very high probability of contagion with more than eight positive cases close to each other. Each point observed in Figure 2, Figure 3, Figure 4 and Figure 5 shows a confirmed case of COVID-19, which are categorized according to their Ct value obtained by qRT-PCR analysis; the red color is indicative of a Ct value < 29, yellow indicates a Ct 30–33, and green is a Ct 34–37.

### 3.2. Risk Area Analysis

To know the risk area, the algorithm weighed the viral load value (Ct) and the proximity between the cases, thus, the different classes observed in Figure 2, Figure 3, Figure 4 and Figure 5 were formed. Similarly, the surface of the urban area of the city of Tepic was measured, which has an approximate extension of 53,180.85 km^2^. For the first wave (Figure 2), the very high-risk area (labeled in red) was 12,276.34 Km^2^ (23.08% of the total urban area). In the second wave (Figure 3), the extension increased to an area of 19,203.08 km^2^ (36.11%), which compared to the first wave, was an increase of 13.03%; while, in the third wave (Figure 4) this same area decreased in size to 10,173.98 km^2^; finally, during the fourth wave the risk area was 16,811.77 km^2^. The sector with a high risk of contagion (in orange) for the first wave (Figure 2) covered an area of 11,141.09 km^2^ corresponding to 20.95%; for the second wave (Figure 3) this was 7891.84 km^2^ covering 14.84% of the urban area, and in the third wave (Figure 4) its extension was 13,227.52 km^2^, covering 24.87%, which compared to the second wave, was an increase of 10.03% (Table 2). The emphasis in these areas is that the development of activities must be carried out with extreme precautionary measures to minimize the risk of transmission and contagion of the SARS-CoV-2.

## 4. Discussion

Since the first cases of SARS-CoV-2 were detected in Tepic, Nayarit (urban area studied in this research) around April 2020 [16], up to 26 January 2022, Nayarit has had 46,664 confirmed cases and this Mexican state has faced a four-pattern epidemiological peak with highest peaks registered in July 2020, the beginning of January 2021, August 2021, and January 2022 [6]. Many measures had been undertaken to mitigate the adverse effects of the COVID-19 pandemic; restrictions on mobility and social distancing, the generalized increase in work at home, the closure of educational and recreational centers, as well as the reduction of close contacts with other people, especially those outside the household [17,18]. The lack of planning, the high cost of PCR analysis, plus the specific characteristics of the pandemic having such as long incubation period, asymptomatic infection, and high false-negative rate diagnosis, have hindered the mitigation of the contagion [19].

According to an estimation proposed by Larremore in 2020, it is proposed that routine monitoring for molecular detection of SARS-CoV-2 infected persons, as well as isolation countermeasures only reduce virus transmission by 30%. In this way, better management of the pandemic requires a multidisciplinary approach and policies strategies, of which testing is only one part [20]. In line with this, spatio-temporal data analysis is noteworthy in multiple time-critical applications [21,22]. Using GIS analysis tools, together with the monitoring of infected persons, it is possible to identify areas at high risk of infection.

The construction of mathematical models applicable to epidemiological phenomena makes it possible to provide estimates and plans based on dynamic, reliable information, with quick access and easy visualization of the effects caused by pandemics. For its part, the identification of areas that can be grouped according to risk provides a solid basis for proposing effective social policies aimed at control and prevention [23]. For this purpose, this study aimed to provide risk maps of SARS-CoV-2 outbreaks in Tepic, Nayarit during four pandemic peaks, risk maps were designed considering the factors of Ct value, population density, and the number of positive cases. As indicated in the literature, these factors are relevant to feed the GIS [24,25,26,27,28,29,30,31,32]. Then risk levels were categorized according to relative density into five classes each, red (very high risk), orange (high risk), yellow (medium risk), green and blue (low risk); and according to viral load into three classes: red (high risk), yellow (medium risk), and blue (low risk).

The proposal for the design of this classification that considers the Ct value as a fundamental factor is based on the work published by Oba and co-workers in 2021 where a Ct value lower than 29 is considered a highly contagious patient who requires strict isolation, and a value of 30 to 33 is considered moderately contagious and quarantine is recommended as a precautionary measure. Finally, with a Ct value between 34 to 37, there is a low risk of contagion and precautionary measures are to maintain the proposed social distancing as well as a second re-examination by qRT-PCR test to observe the evolution of the disease [11]. In this way, the Ct value is very important for the present model because the viral load can also be associated with the infection rate of various respiratory viruses, such as SARS, the influenza virus, and SARS-CoV-2 [9,19,33].

During the analysis of the risk of contagion of the four waves, it became evident that regardless of the area, there is invariably a certain level of risk of viral transmission. Nevertheless, the geographic patterns generated allow discerning in which regions of the city there is a higher risk of transmission; particularly in those areas that appear with high risk in both categories in each wave. These data are essential to help society and decision-makers to enhance health measures. In this context, the characterization of hot spots is only the initial step; once identified, different factors such as the influx of people, urban mobility systems, particulate pollution, the social situation, even meteorological conditions [34], as well as outbreaks of new SARS-CoV-2 variants with a higher rate of infection and evasion of the immune response, such as the delta variant and all others variants of concern (VOC) need to be monitored [35].

The evolution of the COVID-19 pandemic is given by many parameters and variables that give rise to complex phenomena that probably cannot be described or predicted by simple correlations [36]. Although there is no relationship between viral load (Ct value) and age group with respect to SARS-CoV-2 [37], the SARS-CoV-2 infection may be associated with several intrinsic or extrinsic factors (ACE2 expression, co-morbidities, or genetic factors) [38,39,40,41]. Thus, the application of preventive measures, including vaccination, is essential to mitigate the transmission of the disease [30].

In a vaccinated person, the viral clearance will be faster, so there will be lower viral loads, less time of infection as well as a reduction in the probability of developing severe COVID-19 or fatal outcomes [42,43,44]. This is observed in the third wave (August 2021) where most adults were already immunized and therefore the density of infected persons and the viral load (Ct) was lower compared to the first and second waves (Figure 2 and Figure 3). Eventually, as time progresses, the density of infection will decrease because community immunity is being achieved, since adolescents and children are beginning to be vaccinated, thus reducing the number of people that can be infected with SARS-CoV-2 [30].

According to the risk characterization analysis developed for the four waves of COVID-19, it is unmistakable that the extension of the most affected area and its location varied (Figure 2, Figure 3, Figure 4 and Figure 5); however, it can be generally noticed that areas with the most elevated risk of transmission overlapped in zones of high mobility and economic activity since this area includes city downtown, gastronomic corridors, residential areas nearby important shopping centers, and popular flea markets, which are economic centers and present high mobility. These zones were red spots in the risk map during the four maximum peaks of the pandemic. Mobility and socio-economic indicators are closely related as relevant factors in this pandemic as they link human flows and virus transmission rates [18,19]. A blind spot in this study is that economic factors were not used to feed the GIS, this represents an opportunity for future research in the area.

## 5. Conclusions

The use of tools such as GIS to understand the dispersion dynamics of the pandemic in a geographical area is highly relevant for its prediction, prevention, and control. In addition, it is an essential tool to channel efforts in risk areas. Therefore, the proposed model has a great social impact since it can be a useful decision-making tool for the circumscription of high-risk areas. Two factors that our research group considers novel in this type of model are the proximity between cases and especially, the viral load (expressed in Ct value), since this parameter is usually omitted in GIS models, but is a determinant for the potential transmissibility of SARS-CoV-2 between individuals.

## Figures and Tables

**Table 1:** Number of people infected and analyzed through the GIS model during the period established in the four waves of COVID-19.

	Positive Cases	Men	Age Range in Men (yr.)	Women	Age Range in Women (yr.)
First wave: 15 July–14 August 2020	158	81	(17–79)	77	(18–75)
Second wave: 6 January–5 February 2021	254	114	(10–80)	140	(15–85)
Third wave: 4 August–3 September 2021	143	64	(2–73)	79	(2–87)
Fourth wave: 3 January–2 February 2022	279	130	(5–93)	149	(6–82)

yr. = years.

**Table 2:** Area (Km^2^) according to the level of contagion risk in the urban area (total area: 53,180.85 Km^2^) analyzed during the four waves of COVID-19.

Risk Level	First Wave (Km^2^)	Urban Area (%)	Second Wave (Km^2^)	Urban Area (%)	Third Wave (Km^2^)	Urban Area (%)	Fourth Wave (Km^2^)	Urban Area (%)
Very low	8821.08	16.59	9928.73	18.67	10,702.12	20.12	7368.43	13.86
Low	9032.43	16.98	7595.91	14.28	7625.41	14.34	10,554.30	19.85
Medium	11,909.91	22.40	8561.28	16.10	11,451.82	21.53	7274.90	13.68
High	11,141.09	20.95	7891.84	14.84	13,227.52	24.87	11,171.45	21.01
Very high	12,276.34	23.08	19,203.08	36.11	10,173.98	19.13	16,811.77	31.61

Very low corresponds to the blue area on the maps; Low corresponds to the green area on the maps; Medium corresponds to the yellow area on the maps; High corresponds to the orange area on the maps; Very high corresponds to the red area on the maps.

## Data Availability

All data supporting the findings of the study are available with its corresponding author, M.I.G.-P., upon reasonable request.
